# Medullary Thyroid Carcinoma and Papillary Thyroid Carcinoma in the Same Patient as a Collision Tumour

**DOI:** 10.1155/2019/4038628

**Published:** 2019-03-12

**Authors:** Oguz Dikbas, Aslihan Alpaslan Duman, Gulname Findik Guvendi

**Affiliations:** ^1^Giresun University School of Medicine, Department of Internal Medicine, Turkey; ^2^Giresun University School of Medicine, Department of Pathology, Turkey; ^3^Recep Tayyip Erdoğan University School of Medicine, Department of Pathology, Turkey

## Abstract

**Aim:**

Papillary thyroid carcinoma (PTC) and medullary thyroid carcinoma (MTC) are two different types of thyroid carcinoma with significant different clinical and histological findings. Their coexistence in the same patient is a very rare event which demands different clinical approach.

**Case Report:**

We report a case with concurrent MTC and PTC in the same thyroid having characteristics of a collision tumour. A 35-year-old patient has admitted to endocrinology outpatient department with complaint of pain in the neck. Physical examination revealed 2 cm nodule on the thyroid right lobe. Serum thyroid hormone levels were within normal range. Ultrasonography revealed a 23x15 mm hypoechoic nodule with micro calcifications and cystic areas on the right lobe. Preoperative serum calcitonin was 2 pg/ml (0-11.5). PTK 1.7 cm and MTK 1.8 cm in the same thyroid with healthy tissue in between them were detected on pathological examination. RET gene mutation was negative. She has been followed up to now without any evidence of disease.

**Conclusion:**

This is a collision tumour since lesions with features of MTC and PTC were detected in two different locations and separated by normal thyroid tissue. Germline point mutation of the RET gene had a potential role in the development of both MTC and PTC. On the other side, familial concurrent MTC and PTC without RET gene mutation was also published. Both RET and BRAF genes had a role in the development of the medullary and papillary collision tumours. We do not know the presence of BRAF gene mutation in this case report yet.

## 1. Introduction

Thyroid cancer is the most common type of endocrine malignancy with an incidence that has steadily increased for the past three decades [1, 2]. Deaths from thyroid cancers alone account for more deaths than all of the other endocrine malignancies combined in the USA, with an estimate of 62,450 new cases and 1,950 deaths for 2014 [1, 2]. The increased incidence of thyroid cancer diagnoses has been attributed, in part, to improve detection of small or subclinical thyroid nodules by thyroid ultrasonography and other imaging techniques; however, increased incidence of thyroid tumours of all sizes has also been reported [3].

PTC and MTC are two different types of thyroid carcinoma with significant different histology. PTC accounts for the 85% of the differentiated thyroid carcinomas. It is derived from thyroid hormone producing thyroid follicular cells [4]. On the other hand MTC is rare and originates from the calcitonin producing parafollicular C cells which are commonly thought to be derived from the neural crest via the ultimobranchial body. MTC is accepted to be neuroendocrine tumour and secretes calcitonin and other peptide hormones [5, 6]. Synchronous occurrence of these two carcinomas is uncommon and occurs in two different forms: first one is discrete MTC and PTC separated by normal thyroid tissue and the latter one is mixed medullary and follicular-derived thyroid carcinoma. Concurrence of medullary and papillary carcinoma represents less than 1% of all thyroid malignancies [7]. We report a case with concurrent MTC and PTC having characteristics of a collision tumour with a healthy thyroid tissue between them. Clinical course of this kind of tumour is not well known subject.

## 2. Case Presentation

**Figure 1:**
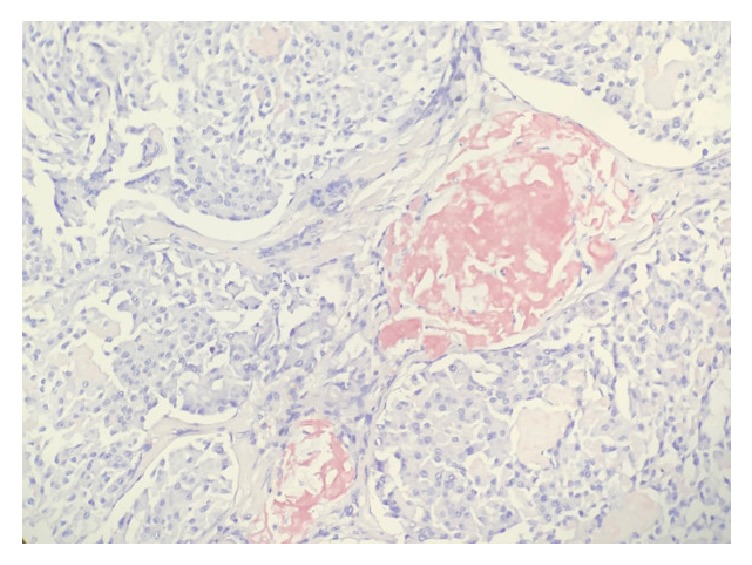
Amyloid accumulation was seen as a pink coloured amorphous material in this section which is typical for medullary thyroid cancer on Congo Red staining.* (20X).*

**Figure 2:**
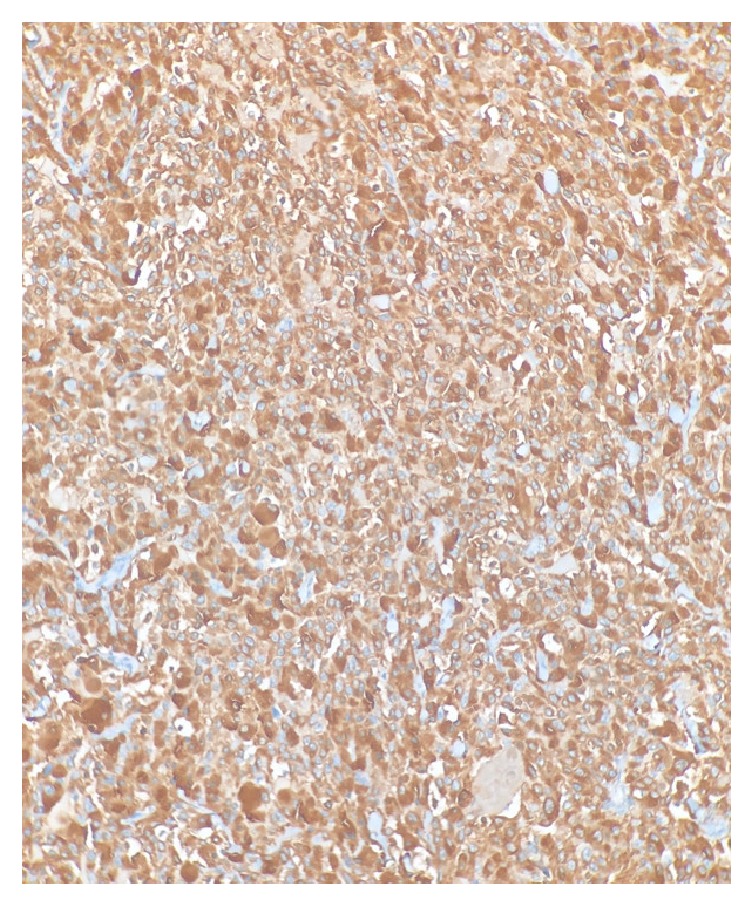
Diffuse cytoplasmic positivity for calcitonin immunohistochemical staining was present in medullary thyroid carcinoma.

**Figure 3:**
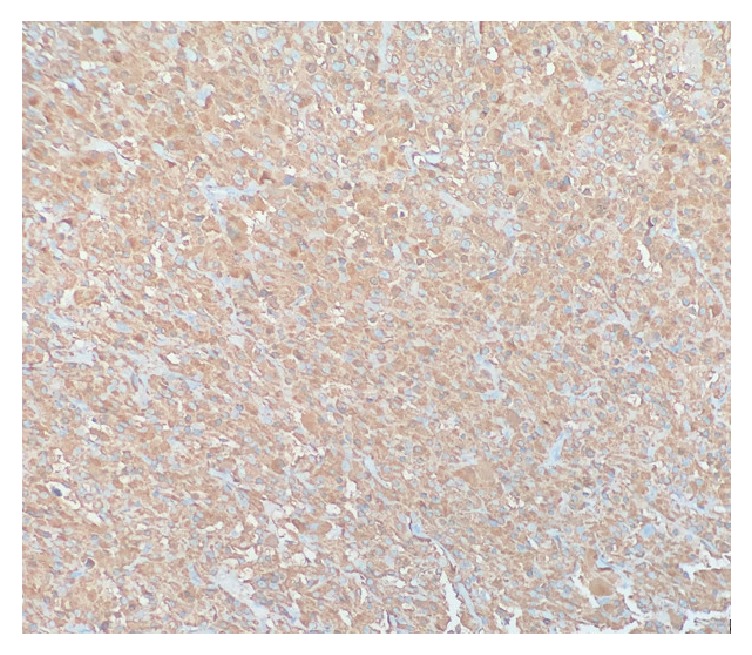
Diffuse cytoplasmic positivity for chromogranin A immunohistochemical staining was present in medullary thyroid carcinoma* (10X).*

**Figure 4:**
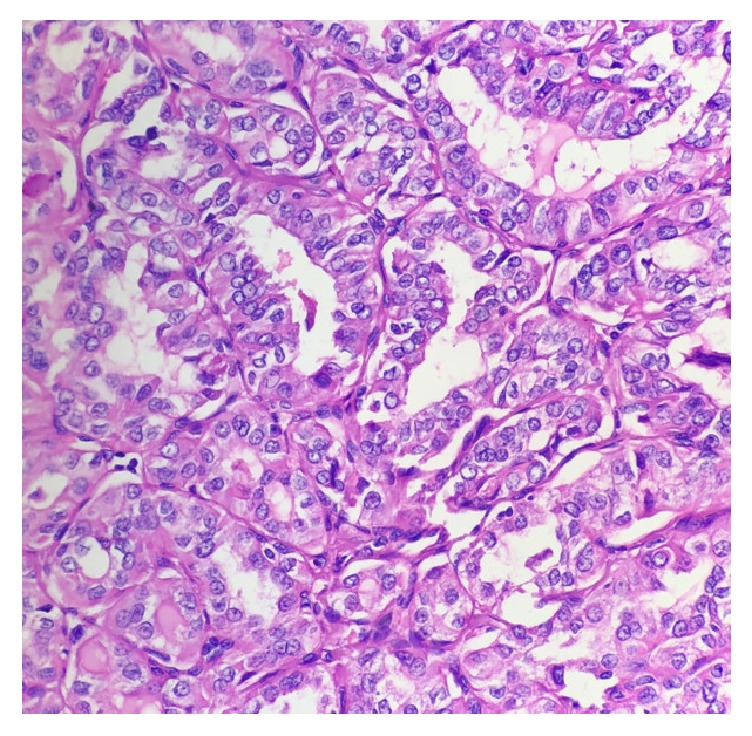
Nuclear grooves with glassy nucleus, peripherally located prominent nucleolus, and nuclear overlapping seen typical for papillary thyroid carcinoma on hematoxylin and eosin staining. H&E*(40X).*

A 35 years old patient has been admitted to endocrinology outpatient department with the complaint of pain in the neck radiating to ears. Physical examination revealed 2 cm nodule on right lobe of the thyroid. She has no personal history of radiation exposure and family history of thyroid cancer. Serum levels of free triiodothyronine and free thyroxine were within normal ranges. Serum calcitonin level was 2 pg/ml (0-11.5) (postsurgical measurement). Antithyroglobulin antibody (anti-Tg Ab) was negative. A preoperative thyrotropin serum value was suppressed (0.129 *μ*U/ml). Thyroid ultrasonography revealed 23 mm hypoechoic nodule with microcalcifications and cystic areas on the right lobe. No pathological lymph node was detected on ultrasonography. Magnetic resonance imaging of the neck revealed no lymph node on the jugular region. Fine needle aspiration biopsy from the thyroid nodule was performed. Prominent polychromasia, overlapping on thyrocytes, and occasional bizarre nuclei were detected on cytology. Primarily it was thought papillary carcinoma of thyroid. Bilateral total thyroidectomy was performed. On pathological examination there were two tumours on the right lobe. These are PTC with 1.7 cm in diameter and MTC with 1.8 cm in diameter. Amyloid-like material accumulation and hemorrhagic stroma were detected on MTC (Figure 1). Also spindle cells with bizarre nuclear structure covered with capsule with local fibrotic areas were seen on pathologic evaluation. Chromogranin A and calcitonin was positive on immunohistochemical staining which was compatible with MTC (Figures 2 and 3). Necrosis, lymphovascular invasion, calcification, and capsular involvement of tumour were not detected in PTC. Papillary component had a capsule. On the light microscopy papillary structures with thyrocytes having nuclear clearing, grooving, and overlapping around fibrovascular core were detected (Figure 4). Remaining thyroid was compatible with lymphocytic thyroiditis. RET gene analysis revealed no MEN 2 related mutation in this patient. She received radio-ablative treatment with I^131^ sixth month later following surgery. She is under suppressive treatment with levothyroxine sodium. She has been followed up without any evidence of disease since then.

## 3. Discussion

Here we present a case having both MTC and PTC in the same thyroid with healthy tissue in between.

MTC may presents as a component of the multiple endocrine neoplasia type 2a (MEN 2A). The gene for the MEN 2 syndromes is the RET proto-oncogene, located at the centrometric region of chromosome 10 (10q11-2). RET proto-oncogene presents sporadically in approximately in 50% [8]. But much of the time it presents sporadically. All classic MEN 2A families studied thus far have germline mutations of the RET proto-oncogene, but a few families with multiple endocrine neoplasia type 2b (MEN 2B) or familial medullary thyroid carcinoma (FMTC) do not have such mutations. ESR2 frame shift mutation has been identified in a FMTC family lacking RET mutation [9]. Other genetic abnormalities identified in patients with classic MEN 2A and FMTC include LOH at chromosomes 1p, 22q, 17p, or 3p. Overall, about 95% of patients with MEN 2 are classified as MEN 2A and 5% as MEN 2B [10]. Indeed, MTC patients with a negative family history need to be screened for MEN 2. These patients were found to have a hereditary syndrome as up to 3-7% [10]. In this case we did not detect any RET mutation. Also there was not any family history of thyroid malignancy.

These tumours were encountered mainly in women as a palpable neck mass. The lymph node metastasis of these tumours shows one of either pathology or combination of them. Distant metastases were seen in lung, liver, and bone. Co-occurrence of MTC and PTC in the same thyroid is a rare phenomenon that can be seen either as a mixed tumour showing dual differentiation or a collision tumour [11–13]. Our case belongs to the latter category since lesions with features of MTC and PTC were detected in two different locations which were separated by a normal thyroid tissue.

The possible histogenetic background and molecular mechanisms that cause mixed medullary-papillary carcinoma of the thyroid are still unclear. The generally accepted hypotheses for coexistence of MTC and PTC are stem cell, collision effect, and hostage theories. Collision effect theory figures the tumour to be composed of two different and asunder moieties. In the most case reports, it was expressed as coincidental or synchronous co-occurrence owing to the separation of PTC and MTC by normal healthy thyroid cells. As RET and BRAF mutations were shown in MTC and PTC components of this tumour, respectively, Rossi et al. proposed the description of “coincidental occurrence” [14]. The term “collision tumour” has been proposed to designate tumours with different genetic and thereby embryologic origin [14].

Tests for the presence of RET oncogene in case reports reveal contradictious results. Germline point mutation of the RET gene had a potential role in the development of both MTC and PTC [15, 16]. It was published that RET and BRAF genes both had a role in the tumorigenesis of the medullary-papillary collision tumours [14]. Cerrato et al. reported that half of sporadic MTCs do not carry RET mutations. Other genes, like retinoblastoma and TP53 tumour suppressor pathways might be involved in MTC tumour genesis [17]. Vantyghem et al. reported eleven cases of familial MTC-PTC, without RET gene mutation [18]. Overall, the molecular evidence gives rise to the hypothesis that both components of these tumours were not derived from a common stem cell [19]. RET oncogene was tested and found to be negative. We do not have any idea about BRAF gene mutation in this patient.

Co-occurrence of MTK and PTK is not common. RET oncogene was tested and found to be negative in this patient. This is a sporadic case which does not require screening for both pheochromocytoma and primary hyperparathyroidism. This case emphasizes measuring serum calcitonin preoperatively which will determine the mode of surgery.
